# Push-Out Bond Strength of Fiber-Reinforced Post Using Various Post Space Irrigation Treatments

**DOI:** 10.1155/2024/8647515

**Published:** 2024-11-06

**Authors:** Amir Mohammad Shahrokhi, Amir Ali Shahrokhi, Raoufeh Asghari, Mehdi Badiee, Kaveh Seyedan

**Affiliations:** ^1^School of Dentistry, Shahid Beheshti University of Medical Sciences, Tehran, Iran; ^2^Dental School, Islamic Azad University of Medical Sciences, Tehran, Iran; ^3^School of Dentistry, Guilan University of Medical Sciences, Rasht, Iran

**Keywords:** fiber posts, post space, root canal irrigant, smear layer

## Abstract

**Objective:** This study aims to provide the impact of different endodontic irrigation treatments after post space preparation on the push-out bond strength of fiber posts to dentin using self-adhesive cements.

**Methods and Materials:** A total of 180 extracted premolar teeth were selected. The canals were instrumented by and then were filled. After preparing the post space, teeth were divided into 12 groups according to irrigants (normal saline solution; 17% EDTA [ethylenediaminetetraacetic acid] + 5.25% NaOCl [sodium hypochlorite]; 17% EDTA; 5.25% NaOCl; and 1.2% chlorhexidine [CHX]) and irrigation technique (conventional syringe irrigation [CSI] and passive ultrasonic irrigation [PUI]). After cementation, the samples were sectioned to obtain 1-mm disks from coronal and apical parts, a push-out test was performed using a universal testing machine (Z050, Zwick/Roell, Ulm, Germany) at a speed of 1 mm/min. The data were analyzed using one-way analysis of variance (ANOVA) and Mann–Whitney U tests.

**Results:** The push-out bond strength was higher in coronal segment in comparison to apical segment. The highest push-out bond strength value was related to NaOCl + EDTA using PUI (12.39 ± 1.08). There are significant differences when comparing NaOCl + EDTA group with EDTA group using CSI or PUI technique (*p*  < 0.05) except in coronal region with CSI technique. In both apical and coronal regions, phosphoric acid irrigation was found to be similar to normal saline solution and CHX groups regardless of activation technique.

**Conclusion:** The push-out bond strength values were significantly affected by the irrigation protocol. Using PUI and 17% EDTA + 5.25% NaOCl in apical region could be an effective irrigant for smear layer removal after post space preparation.

## 1. Introduction

In nature, dental hard tissues are rarely self-repairing. It is revealed that endodontic treatment of teeth removes much of the coronal tooth structure, which may present a high risk of biomechanical tooth failure [1]. However, there is a controversy surrounding the decision to place the post [1, 2]. Among other options, fiber posts have gained popularity in dental practice due to their ability to uniform force distribution along the tooth–post interface, reducing stress concentration into the dentin [3–5]. The bond strength of fiber-reinforced posts to root dentin can vary depending on factors, such as the type of glass–fiber post system, the location of prepared post spaces, number of remaining walls, close adaptation of cement into dentinal tubules, irrigation solution, and conditioning technique [1–5]. Despite the fact that they possess various advantages, one drawback of fiber-reinforced posts is their debonding and the most common adhesive bond failure occurs at the resin–dentin interface [6].

Preparation of post space results in heavy smear layer deposits on dentine walls. This may act as a barrier and affect the depth to which intracanal adhesive can penetrate dentinal tubules, thereby reducing treatment success [7]. Post space irrigation (PSI) may influence the strength of the cement bond within the root canal system. Irrigants features include organic or inorganic tissue-dissolving ability or antimicrobial/antibiofilm activities. The most common irrigation agent for removing the inorganic part of the smear layer is ethylenediaminetetraacetic acid (EDTA), which refers to the chelating agent [8]. Although it can effectively remove the smear layer produced during canal instrumentation. Sodium hypochlorite (NaOCl) is another commonly used irrigant, with concentrations ranging from 1.3% to 5.25% [9]. Researchers have also reported that the combination of NaOCl and EDTA effectively reduces canals bacteria [8, 10]. Due to its ability to participate and coagulate bacterial intracellular constituents, chlorhexidine (CHX) is known for its antimicrobial properties and can be used as an alternative to NaOCl [11]. Phosphoric acid at 37% is a conditioning substance that showed favorable results because of its effectiveness in removing the smear layer [12].

Not only the properties of irrigation solution but also the delivery system used for irrigation is critical in efficacy of irrigation. Various techniques for irrigation delivery and activation, including conventional syringe irrigation (CSI) and passive ultrasonic irrigation (PUI), have been advocated for the root canal debridement [13]. However, they have their own pros and cons.

This research paper aims to provide a comprehensive understanding of the impact of different endodontic irrigation treatments after post space preparation, including 37% phosphoric acid, 5.25% NaOCL, EDTA, 1.2% CHX, and 0.9% saline solution with and without PUI, on the push-out bond strength of fiber posts to dentin using self-adhesive cements.

## 2. Materials and Methods

### 2.1. Sample Preparation

The protocol of this in vitro experimental study was approved by the ethics committee of the Research Council of School of Dentistry, Shahid Beheshti University of Medical Sciences (IR.SBMU.DRC.REC.1398.138). In this study, 180 sound human single-rooted maxillary second premolars extracted for periodontal or orthodontic reasons were selected and examined using a stereomicroscope (SZX 16; Olympus, Tokyo, Japan) to ensure they were free of fractures, restorations, caries, calculus, and any other abnormalities. The specimens were disinfected in a 0.5% chloramine T solution for 1 week at 4°C. Throughout the experimental procedures, they were maintained in distilled water at 37°C.

Standard access cavities will be prepared using a high-speed handpiece (Midwest Dental Supply, Des Plaines, IL, USA) with air–water cooling. The working length was determined as 1 mm short of the foramen. The root canals were shaped using ProTaper Gold files (Dentsply, Charlotte, NC, USA) up to F3 (size 30, 0.09 taper). Irrigation was involved with 10 mL of 5.25% NaOCl, and the smear layer was removed using 17% EDTA for 1 min. The canals were then filled using gutta-percha points and AH-Plus sealer (Dentsply Sirona, Tulsa, OK, USA) through lateral compaction. Subsequently, the crown was removed by utilizing a high-speed diamond disc (Drendel and Zweiling [D&Z], Darmstadt, Germany) with water coolant. For post space preparation, size 3 gates glidden drill followed by ParaPost drill size 3 (Coltene/Whaledent, Altstätten, Switzerland) was used to remove gutta-percha.

### 2.2. Irrigation Treatments

After post space preparation, samples randomly were divided into 12 groups (*n* = 15) according to CSI or PUI. The specimens were treated with various irrigants, including phosphoric acid (BISCO Inc., Illinois, USA), 5.25% NaOCl (Cerkamed, Stalowa Wola, Poland), 17% EDTA (Cerkamed, Stalowa Wola, Poland), and 1.2% CHX (Cerkamed, Stalowa Wola, Poland), as outlined in Table 1. After irrigation, paper points were used to dry the root canals. All procedures were conducted by the same operator.

### 2.3. Assessment of Push-Out Bond Strength

The fiber posts were cemented using the light finger pressure by Theracem (BISCO Inc., Illinois, USA) self-adhesive resin cement following the manufacture instructions. The specimens were subsequently incubated in distilled water for 24 h at 37°C to allow for complete polymerization. After incubation period, samples were embedded in an autopolymerizing acrylic resin. The resulting blocks were then sectioned using low-speed disc with water coolant, yielding two slices from apical and coronal region of tooth–post complex. Each slice of the sample was standardized to thickness of 1 mm.

Push-out bond strength values were measured using a universal testing machine (Z050, Zwick/Roell, Ulm, Germany) at a speed of 1 mm/min using 1 mm diameter metallic plunger in coronal third, and 0.5 mm in apical third portion from apical to coronal direction in all samples until the post dislodges from the tooth. The maximum load applied to the materials was recorded in Newton (N) before dislodgment occurred and to express the data in MPa, the recorded value in N was divided by area in 2 mm calculated by the following formula: 2*πrh*, where *π* is the constant 3.14, *r* is the root canal radius, and *h* is the thickness of the root slice in mm.

### 2.4. Statistical Analysis

Statistical analyses were conducted using SPSS software version 26. A one-way analysis of variance (ANOVA) was applied to compare the index across the 12 groups for both apical and coronal sections. The Games–Howell test was used as an appropriate post hoc test for pairwise comparisons. To compare PUI versus CSI and divide the 12 groups into categories for coronal and apical sections, the nonparametric Mann–Whitney U test was used. A *p*-value of 0.05 was set as the level of significance for all statistical tests.

## 3. Results

The mean and standard error of all groups are displayed in Table 1. Mann–Whitney U test demonstrated that in both coronal and apical regions, there is a significant difference between CSI and PUI techniques (*p*=0.001). The highest push-out bond strength value was observed in the apical region with NaOCl + EDTA using PUI (12.39 ± 1.08), while the lowest was found in apical region with EDTA using CSI (3.04 ± 0.51).

Multiple comparisons in both coronal and apical regions revealed that there are no significant differences between NaOCl + EDTA in comparison with NaOCl using CSI or PUI technique (*p*  > 0.05). However, there are significant differences when comparing NaOCl + EDTA group with EDTA group using CSI or PUI technique (*p*  < 0.05) except in coronal region with CSI technique. The results in normal saline solution group showed that CSI technique significantly obtained higher value than PUI group in coronal region (*p*=0.007). In both apical and coronal regions, phosphoric acid irrigation was found to be similar to normal saline solution and CHX groups regardless of activation technique.

## 4. Discussion

The push-out bond strength test was first advocated by Roydhouse [4]. It is a more predictable method for determining bond strengths, as it results in true shear stress that is comparable with the stress under clinical conditions [4]. Therefore, a push-out bond strength test was performed in the present study. The results demonstrated that different irrigation methods cause changes in the pushout bond strength of fiber post to dentin, leading to the rejection of the null hypotheses.

Our results indicated that coronal regions had a slightly greater push-out bond strength compared to apical regions in most groups. This variation in bond strength can be attributed to structural differences including descending density of the radicular dentin sections toward the apex [14]. This affects the ratio between peritubular and intertubular dentin, compared to the coronal segment. Some studies [14–16] reported that C-factors, limited access, and reduction in the hybrid layer thickness in apical part of root could be also contribute to lower bond strength in this area.

According to the result of this study, in normal saline solution group, CSI technique significantly obtained a higher value than PUI group in the coronal region. It is clear that saline solution lacks antimicrobial and chelating properties and while the cleaning efficacy of PUI is well documented for effective removal of dentin debris, microorganisms (planktonic or in biofilm), and organic tissue from root canal [17], its impact on the bonding ability of radicular dentin to fiber post is not well understood and requires further investigation. The challenge of obtaining a uniform cleaning of the root dentinal surface has led to controversial results.

The highest push-out bond strength value was observed in NaOCl + EDTA group using PUI which is in line with some studies [18, 19] that reported significant improvement in smear layer removal efficacy with NaOCl + EDTA irrigation in the post space. EDTA acts as a chelating agent leads to dentine demineralization and leaves the collagen scaffold exposed. Irrigating this exposed surface with 5.25% NaOCl causes the collagen dissolution. Therefore, these two agents could have a complete effect on organic and inorganic components of the smear layer. It is also indicated that the PUI technique promotes better cleaning of the root canals as it increases the flow of the solution and the contact of the irrigating solution with the dentinal walls and creates a flow-reflux mechanism that drives the debris out of the root canal [17, 18].

According to the other result related to NaOCl or EDTA, only regardless of CSI and PUI, it appears that EDTA is not able to completely remove smear layer because of decreasing pH during demineralization and self-limitation effect [20]. The lower bond strength in EDTA irrigation group could be attributed to complete demineralization of collagen fibers and reduced chemical bonding process of self-adhesive cement.

CHX is an matrix metalloproteinases (MMPs) inhibitor that protects the collagen from degradation in hybrid layer leading to higher bond strength. The nonsignificant differences from normal saline group could be demonstrated that short-term evaluation may influence the results. These results are in consistent with Ali and Kadhim's [17] study. In addition, Abbasi et al. [21] believed that root dentin preparation with 37% phosphoric acid eliminates the smear layer formed during endodontic treatment or post space preparation and opens dentinal tubules, yielding a clean surface. So, may increase the bond strength. Contrary to our result, phosphoric acid did not improve the bond strength because the effect of surface treatment-resin cement type relationship on bond strength can be more complex. Different resin cements can perform differently in similar surface treatments. The complex structures should be investigated in further studies.

This study has several limitations. First, the study was conducted on extracted teeth, which may not be representative of the clinical situation. Second, the study used a single type of fiber post and a single type of self-adhesive cement. The results may not be generalizable to other types of fiber posts or self-adhesive cements. Third, the study did not assess the long-term durability of the bond strength. Further studies are required to investigate the long-term durability of the bond strength of fiber posts luted with self-adhesive cements using different irrigation protocols.

## 5. Conclusion

The push-out bond strength values were significantly affected by irrigation protocol. PUI had a nonsignificant effect on bond strength of fiber post in apical region in comparison with coronal region. The push-out bond strength was higher in coronal segment in comparison to apical segment. It can be concluded that using PUI and 17% EDTA + 5.25% NaOCl in apical region could be an effective irrigant for smear layer removal after post space preparation.

## Figures and Tables

**Table 1:** The mean ± SE (MPa) push-out bond strength between groups at apical and coronal region.

Irrigation technique	Irrigation treatment	Mean ± SE (MPa)	
		Coronal	Apical
CSI	Normal saline solution	8.40 ± 0.79	7.20 ± 0.81
	Phosphoric acid	7.22 ± 1.20	4.75 ± 0.92
	NaOCl	5.55 ± 0.80	3.62 ± 0.64
	EDTA	5.54 ± 1.23	3.04 ± 0.51
	NaOCl + EDTA	8.06 ± 0.70	7.50 ± 1.02
	CHX	8.38 ± 1.38	5.09 ± 1.06

PUI	Normal saline solution	4.33 ± 0.36	4.28 ± 0.21
	Phosphoric acid	7.04 ± 1.83	5.89 ± 0.62
	NaOCl	9.67 ± 1.25	9.33 ± 1.02
	EDTA	4.80 ± 0.76	5.69 ± 0.81
	NaOCl + EDTA	9.62 ± 0.87	12.39 ± 1.08
	CHX	8.88 ± 1.22	5.68 ± 1.03

Abbreviations: CHX, chlorhexidine; CSI, conventional syringe irrigation; EDTA, ethylenediaminetetraacetic acid; NaOCl, sodium hypochlorite; PUI, passive ultrasonic irrigation; SE, standard deviation.

## Data Availability

The data used to support the study are included in the paper.
